# 

**Published:** 1918-05-25

**Authors:** 


Rates of Subscription: Twelve months, 6/6; Six months, 4/-; three months, 2/-, post free to any address in the United Kingdom. Abroad, Twelve months, 8/8; Six months, 5/-; Three months, 2/6, post free. THE HOSPITAL "Index" to Volume LXIII. October 1917 to March 1918, is now ready, price 3½d. per copy, post free. Address The Manager, The Hospital, "The Hospital" Building, 28/29 Southampton Street, Strand, London, W.C. 2.

### TUBERCULOSIS TOPICS.

It is always encouraging to see a right decision come to, and the right thing done, against difficulties. At a recent meeting of the Middlesex Health Insurance Committee there came up for consideration the Sanatorium Sub-Committee's report, which mentioned a waiting-list of sixty-nine tuberculous patients, including men from the Forces. To provide further institutional accommodation it was therefore recommended to petition the National Health Insurance Commissioners and the Local Government Board, firstly, for assistance in extending the Clare Hall Hospital by forty beds, and, secondly, to sanction the County Council to take over liability for the treatment of cases of tuberculosis in Poor-Law institutions. The latter proposal met with some opposition. Sentimental considerations were urged against sending patients to Poor-Law infirmaries, a resort which was excluded in the original framing of the Act. It was argued, too, that the provision of huts would meet the case. Unfortunately a medical man was found to back the proposal for huts. But ultimately good sense triumphed, and the accommodation ready to hand, in the shape of Poor-Law buildings, was accepted, a most fortunate decision at a time when all building operations, even of an elementary kind, for non-military purposes are necessarily at a standstill. This discussion illustrates many of the defects of anti-tuberculosis administration in this country. A practical tuberculosis physician, one with sanatorium experience, could have told of the failure of the hut system on a large scale for working-class patients. In winter the hardships are such that very many, especially discharged soldiers, will not stand them. Huts mean cold meals for bed patients, delay in treating hæmoptysis, increased work for the staff. They mean, too, great difficulty in supervision; after nightfall patients can easily slip away and get into mischief. But with the status of the resident sanatorium doctor what it is in this country, with his practical exclusion from any authoritative standing and voice, the right direction of the British anti-tuberculosis movement will continue to be largely a matter of luck.

Sir Robert Philip's inaugural address on his appointment to the Edinburgh Chair of Tuberculosis was on well-known lines. A member of the "contagionist" school, he looks to segregation and treatment of existing cases as the main means of combating the disease, and considers that its extinction is hopeful and, indeed, a mere matter of applying such means thoroughly enough. Unfortunately, as pointed out a little while back in a leading article in the *Lancet*, this view has been shaken by the phenomenon of a great increase in tuberculosis occurring since the war began. Such increase has been much too sudden to be attributable to greater facilities of infection, and is agreed to be due to impairment, on a large scale, by many war conditions, of the general health of the nations at war. Besides this, there is the as yet practically unconsidered factor of intrinsic predisposition, long suspected, but hitherto little defined. In short, it is the old tale, everywhere renewed, of etiology being a much more complex affair than at first apparent; of becoming the more complex with each step in its elucidation.

### Forthcoming Events.

**COLLEGE OF NURSING (Limited by Guarantee).** —Conference, June 6 and 7, at the Medical Society of London, 11 Chandos Street, Cavendish Square, W. **Thursday, June 6, 7.30 p.m.**—"The Higher Education of Nurses with Relation to Applicants for Training," by Miss Gullan, Sister-Tutor of St. Thomas's Hospital; Surgeon-General Sir Berkeley Moynihan, F.R.C.S., in the chair. **Friday, June 7, 11 a.m.**—"The Advantages and Disadvantages of a Preliminary Training for Intending Probationers," and "The Payment of Nurses in Training," by Miss A. Margaret Cummins, Matron, Royal Infirmary, Liverpool; Lieut.-Col. D'Arcy Power, F.R.C.S., in the chair. **3 p.m.**—"The Position of the Trained Nurse in Relation to the Work of Social Reconstruction"; Miss Haldane in the chair.

Royal Hospital and Home for Incurables, Putney Heath.—War-time Luncheon at Grocers' Hall, Princes Street, E.C., Thursday, June 6, at 1 for 1.30 precisely, the Right Hon. the Lord Mayor of London in the chair.

Asylum Workers' Association.—Annual meeting at the Mansion House, London, E.C., Wednesday, May 29, 1918, at 3 p.m., the Rt. Hon. The Lord Mayor (Vice-President of the Association) in the chair.

### A Joint Appeal for Orphanages.

The Lord Mayor has opened a Mansion House Fund for the financial assistance of seven of the principal orphanages—namely, the Royal British Orphan Schools, Slough; the Brixton Orphanage for Fatherless Girls; the Home for Female Orphans, St. John's Wood; the Royal Female Orphan Asylum, Beddington; the Infant Orphan Asylum, Wanstead; the Orphan Working School, Haverstock Hill; and the Reedham Orphanage at Purley.

### Matrons' and Sisters' Appointments.

Auxiliary Military Hospital, Cliffden, Saltburn-by-the-Sea.—Miss E. A. Hextall has been appointed matron. She was trained at the General Infirmary, Burton-on-Trent, has been on active service in France, sister-in-charge of the Filey V.A.D. Hospital, Filey, and matron of the Auxiliary Military Hospital, Market Drayton. She has also done private nursing.

Brook House Auxiliary Military Hospital, Levenshulme.—Miss E. M. Ault has been appointed matron. She was trained at Wakefield General Hospital, and has since been sister at the Victoria Hospital, Tynemouth, at the General Hospital, Stratford-on-Avon, at the Princess Alice Hospital, Eastbourne, sister and matron of Meathop Sanatorium, Grange-over-Sands, and matron of Mesh Hall Auxiliary Military Hospital, Southport.

Fusehill War Hospital, Carlisle.—Miss S. G. Dalziel has been appointed sister. She was trained at the Kilmarnock Infirmary, Ayrshire, and has been charge nurse at the City Hospital, Hull. She has been a member of the Territorial Force Nursing Service, serving in the 1st Northern Hospital, Newcastle, in Manchester, and in Salonika.

Kent and Canterbury Hospital, Canterbury.—Miss Alberta Chart has been appointed sister. She was trained at the Nelson Hospital, Merton, has been staff nurse at St. Mary's Hospital, Plaistow, at the Norfolk War Hospital, Norwich, ward and theatre sister at Hill House War Hospital, Minster, and ward sister at the Hôpital Anglais, Rhône, France.

Longmore Hospital for Incurables, Edinburgh.—Miss Mary Archibald has been appointed night sister. She was trained at the Western District Hospital, Glasgow, and also holds the certificate of the Central Midwives Board. She has since been assistant matron of Dykebar Asylum, Paisley, sister at Norwich War Hospital, and night sister at Perth War Hospital. Miss Archibald has also worked in Canada.

Norwich Infirmary.—Miss Ellen Farman has been appointed night superintendent. She was trained at Tonbridge Union Infirmary, and has since been staff nurse and sister at North Evington Infirmary, Leicester; pupil midwife at the Louise Margaret Hospital, Aldershot, where she gained the certificate of the Central Midwives Board; senior day charge nurse at Epsom Infirmary and at Colchester Infirmary, and night charge nurse at Yarmouth Infirmary. Miss Grace E. Page has been appointed ward sister. She was trained at Kensington Infirmary, where she also gained the Central Midwives Board certificate, and later became ward sister. Miss Emily Hathaway has been appointed ward sister. She was trained at Norwich Infirmary, where she was later staff nurse, and where she gained the C.M.B. certificate.

No. 4 War Hospital, Reading.—Miss Gough has been appointed matron. She was trained at Bristol Royal Infirmary, and has since been charge nurse at the Park Hospital, Lewisham, sister at Birmingham General Hospital, and matron of Surbiton Hospital, of Torquay Auxiliary Military Hospital, and of the Dorset County Home for Nurses.

Rosehill Children's Hospital, Torquay.—Miss Norah Thorpe has been appointed sister. She was trained at the Children's Hospital, Paddington Green, London, where she has taken sister's holiday duty, and has done private nursing.

Sir Titus Salt's Hospital, Shipley, Yorks.—Miss S. A. Rogers has been appointed matron. She was trained at Bradford Royal Infirmary, and has been sister at Sir Titus Salt's Hospital for eleven years.

**NOTE.—Particulars of Appointments for insertion in this column will be welcomed.**

